# Short term detection of de novo gastroesophageal reflux disease after laparoscopic sleeve gastrectomy

**DOI:** 10.1186/s12876-026-05018-7

**Published:** 2026-07-14

**Authors:** Mostafa Mamdouh Mohamed Abdel-Salam, Alaa Abbas Sabry Moustafa, Ahmed Helmy Youssef, Moheb Shoraby Eskandaros

**Affiliations:** Department of General Surgery, Faculty of Medicine, Ain Shams University, Cairo, Egypt

**Keywords:** Laparoscopic sleeve gastrectomy, Gastroesophageal Reflux Disease (GERD), Bariatric surgery, Obesity, Postoperative complications

## Abstract

**Background:**

The escalating global prevalence of obesity presents a significant public health concern, driving increased rates of obesity-associated comorbidities. Timely identification and management are essential to reduce long-term health and economic consequences.

**Objective:**

This study sought to evaluate the short-term emergence of gastroesophageal reflux disease (GERD) in patients with severe obesity undergoing laparoscopic sleeve gastrectomy (LSG) early in the first 6 months postoperatively.

**Methods:**

We conducted a prospective case series involving 60 patients with severe obesity who received LSG at the Bariatric Surgery Unit, Department of Surgery, Ain Shams University Hospitals (June 2018—June 2021). Postoperative follow-up included clinical evaluation and diagnostic testing to detect new-onset GERD.

**Results:**

GERD developed in 26.7% of patients (16 of 60) following LSG. Based on these findings, LSG should be avoided in individuals with preoperative evidence of erosive esophagitis, Barrett’s esophagus, or an incompetent lower esophageal sphincter (LES), where Roux-en-Y gastric bypass may represent a more suitable alternative. For selected patients with a BMI of 30–35 and associated comorbidities, LSG combined with anti-reflux procedures or concurrent hiatal hernia repair could be considered.

**Conclusion:**

The study reinforces the need for thorough preoperative evaluation, including routine upper gastrointestinal endoscopy, to screen for GERD, hiatal hernia, and premalignant conditions. Careful patient selection and individualized surgical planning remain critical to optimizing outcomes after bariatric surgery.

**Supplementary Information:**

The online version contains supplementary material available at 10.1186/s12876-026-05018-7.

## Introduction

Obesity presents a significant worldwide health challenge, significantly contributing to metabolic, cardiovascular, and neoplastic disorders that detrimentally impact both longevity and quality of life. As non-surgical interventions often yield limited long-term efficacy, bariatric surgery has emerged as the definitive therapeutic approach for severe obesity, offering durable weight reduction, resolution of associated comorbidities, and substantial long-term health improvements [1, 2].

Laparoscopic sleeve gastrectomy (LSG) has become increasingly prevalent among contemporary bariatric procedures, recognized for its favorable safety profile and effectiveness in managing severe obesity [3]. The operation entails excising a large portion of the stomach to form a restrictive tubular conduit. Crucially, LSG maintains normal intestinal continuity, thereby minimizing the potential for malabsorptive complications and micronutrient deficiencies compared to bypass techniques [4].

Gastroesophageal reflux disease (GERD) ranks among the most common chronic digestive disorders globally, with recurrent symptoms like heartburn affecting up to 30% of adults in certain populations. Obesity constitutes a well-established risk factor, with clinical GERD manifestations observed in nearly half of individuals with severe obesity [5]. A considerable proportion of bariatric surgery candidates report pre-existing upper gastrointestinal symptoms, including epigastric pain (28%) and postprandial distress (11%). LSG fundamentally alters upper gastrointestinal anatomy and physiology through resection of the gastric fundus, corpus, and partial antrum. These changes influence gastric compliance, acid secretion, and motor function, potentially impairing accommodation and predisposing patients to new or worsened postoperative symptoms, notably GERD [6].

Resection extent, particularly near the antrum where the gastric pacemaker resides, may further disrupt normal motility patterns. The resultant reduction in gastric reservoir capacity can elevate intraluminal pressures within the neo stomach, contributing to dyspeptic complaints [6]. Technical execution during sleeve creation is paramount. Distortion of the gastroesophageal junction, alteration of the angle of His, or subtle narrowing at the incisura angularis may create functional stenoses. Such alterations increase intragastric pressure, elevating the risk of new-onset GERD. Procedural variability, especially when performed by multiple surgeons, can further exacerbate these technical risks [7].

Sometimes the remaining antrum or the narrowed sleeve creates a functional delay, slowing the passage of solids. A gastric emptying half-time exceeding 21 min is significantly correlated with increase GERD postoperatively [8].

### Objectives

To assess the short-term incidence and characteristics of GERD in severe obese patients following LSG in the early post-operative period (within 6 months).

## Materials and methods

A prospective case series was carried out at the Bariatric Surgery Unit, Department of Surgery, Ain Shams University Hospitals, over a three-year period from June 2018 to June 2021. The study enrolled 60 patients with severe obesity who were selected based on established eligibility criteria and subsequently underwent LSG.

### Patient selection

This study enrolled adult patients meeting established criteria for bariatric surgery: body mass index (BMI) ≥ 40 kg/m^2^, or BMI ≥ 35 kg/m^2^ with significant obesity-related comorbidities, following unsuccessful non-surgical weight management efforts (e.g., structured dietary modification). Participants were self-reported volume eaters without predominant sweet preference.

All eligible candidates received comprehensive preoperative counseling detailing available surgical interventions for severe obesity. This included explicit discussion of the anticipated benefits, procedural risks, and potential complications specific to laparoscopic sleeve gastrectomy (LSG). Written informed consent was obtained from every participant prior to inclusion.

### Inclusion criteria

Patients were included if they met the following criteria: Age between 18 and 60 years, BMI ≥ 40 kg/m^2^ or BMI ≥ 35 kg/m^2^ with documented obesity-associated comorbidities, absence of endocrine disorders as the primary etiology of obesity, deemed psychologically suitable and motivated for surgery and lifestyle changes, no documented history of preoperative GERD or hiatal hernia (confirmed by preoperative endoscopy), provided written informed consent indicating understanding and acceptance of surgical risks and demonstrated commitment to adhering to scheduled long-term postoperative follow-up assessments.

### Preoperative assessment

All patients underwent a comprehensive preoperative evaluation to assess their suitability for surgery. This included a thorough medical history review and clinical examination. Laboratory investigations comprised CBC, liver and kidney function tests, liver enzymes, fasting blood glucose, coagulation profile, thyroid function tests (T3, T4, and TSH), and lipid profile. Hormonal evaluation was performed to exclude endocrinological causes such as hypothyroidism. Pulmonary assessments included chest X-ray and pulmonary function tests. Radiological imaging involved abdominal ultrasonography to detect gallstones or other abnormalities, with patients showing such findings excluded from the study. Cardiac evaluation included an electrocardiogram (ECG), and when indicated, echocardiography and duplex ultrasound of the lower limbs. Additionally, all patients underwent esophagogastroduodenoscopy (EGD) to rule out GERD, esophagitis, Barrett’s esophagus, and hiatal hernia. The goal of this extensive preoperative workup was to ensure surgical eligibility, optimize the management of comorbidities, and provide comprehensive education to both patients and their families about the procedure, potential risks, and postoperative expectations.

### Surgical preparation

Unless medically indicated otherwise, patients were typically admitted on the day of surgery and targeted for discharge within 48 to 72 h if no complications arose. Preoperative preparation involved detailed counseling and obtaining informed consent from each patient. Preventive measures against deep vein thrombosis (DVT) included administration of prophylactic enoxaparin and the use of compression stockings. Additionally, all patients received preoperative antibiotics, specifically third-generation cephalosporins as a local hospital policy and had Foley catheters inserted to monitor urine output during surgery.

### Operative technique

All procedures were performed using a standardized laparoscopic sleeve gastrectomy technique by two experienced bariatric surgeons. Patients were placed in the supine reverse Trendelenburg position, with the surgeon positioned between the legs.

**Fig. 1:**
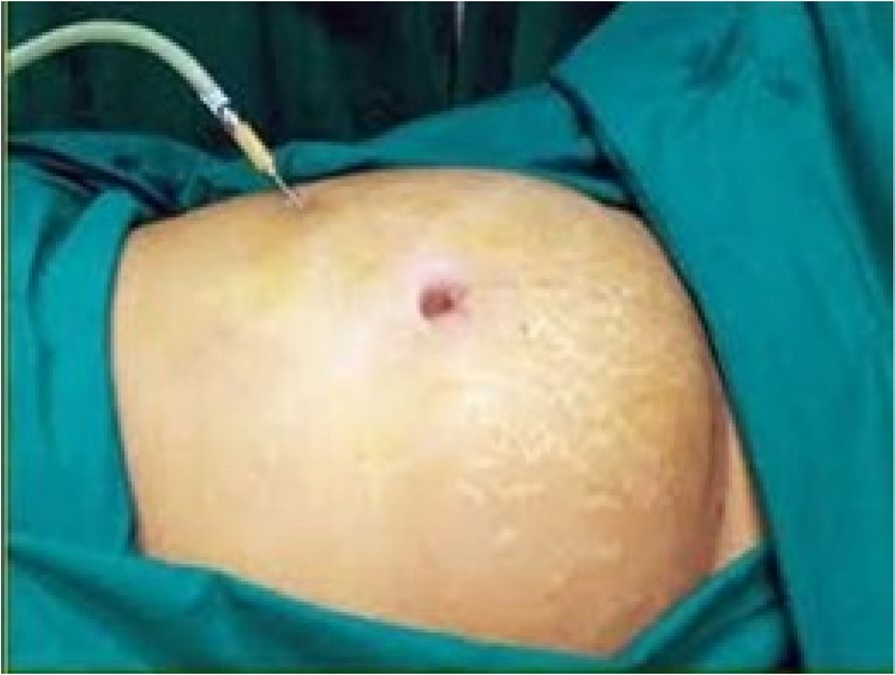
Veress needle insertion

**Fig. 2:**
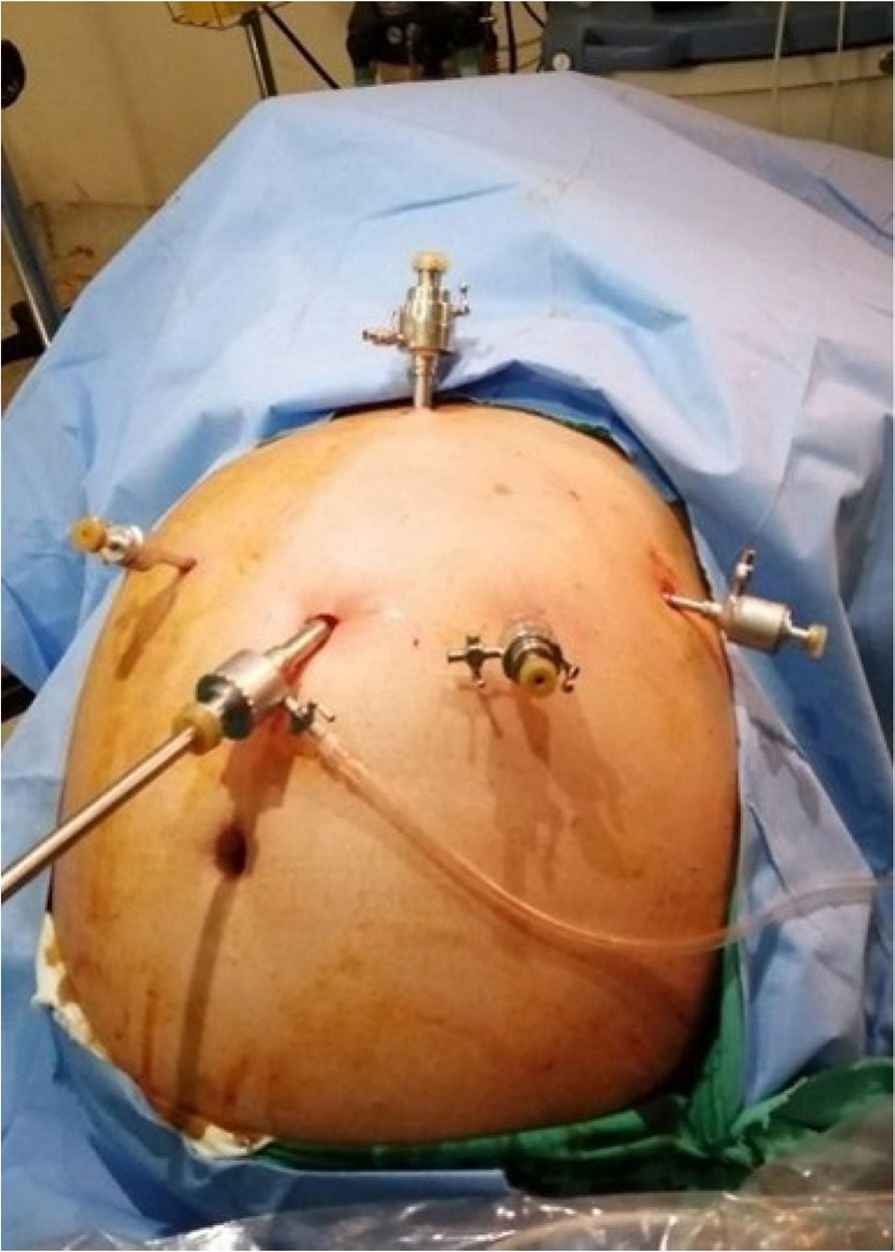
Patient in the operating room with port placement

**Fig. 3:**
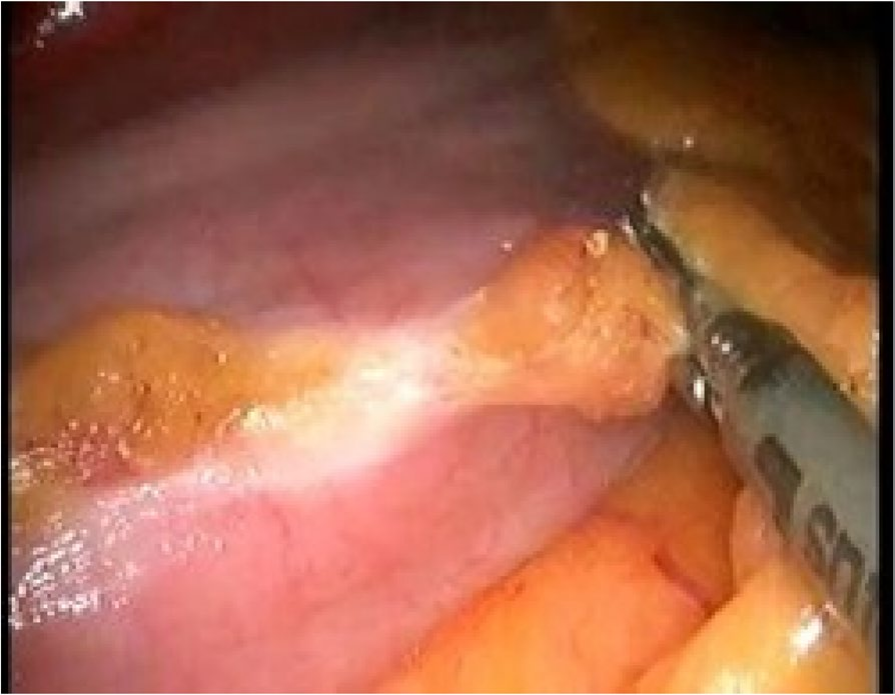
Freeing of the greater curve from the greater omentum

Pneumoperitoneum (15 mmHg) was established using a Veress needle at Palmer’s point (Fig. 1). A standardized five-port technique was used (Fig. 2). A 36 Fr orogastric bougie was introduced to calibrate the gastric sleeve (Fig. 3).

**Fig. 4:**
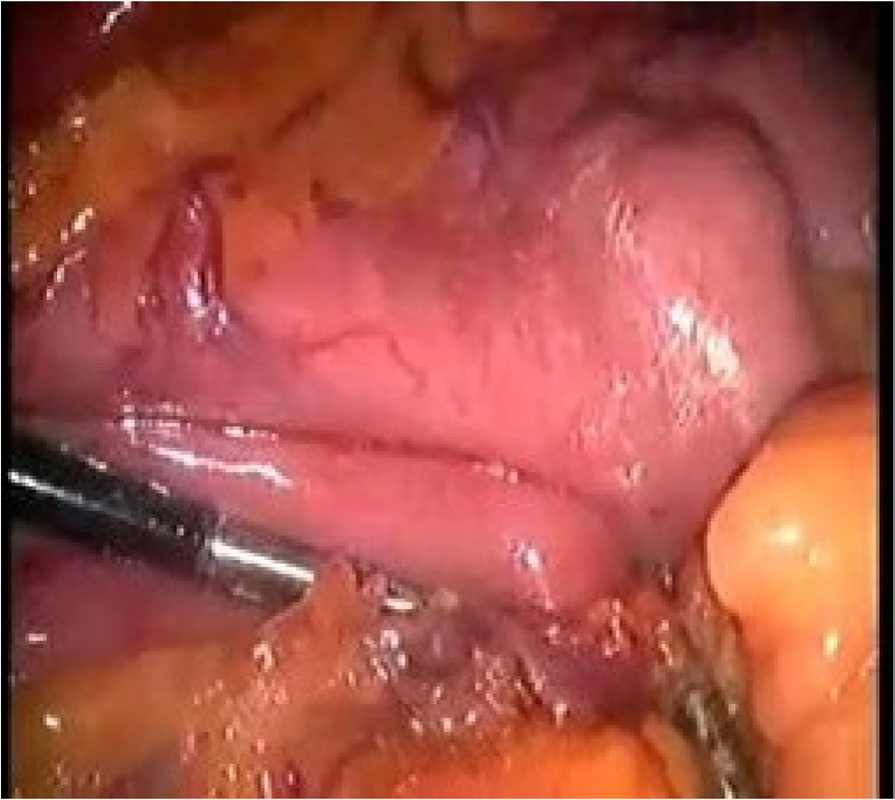
Introduction of the bougie

**Fig. 5:**
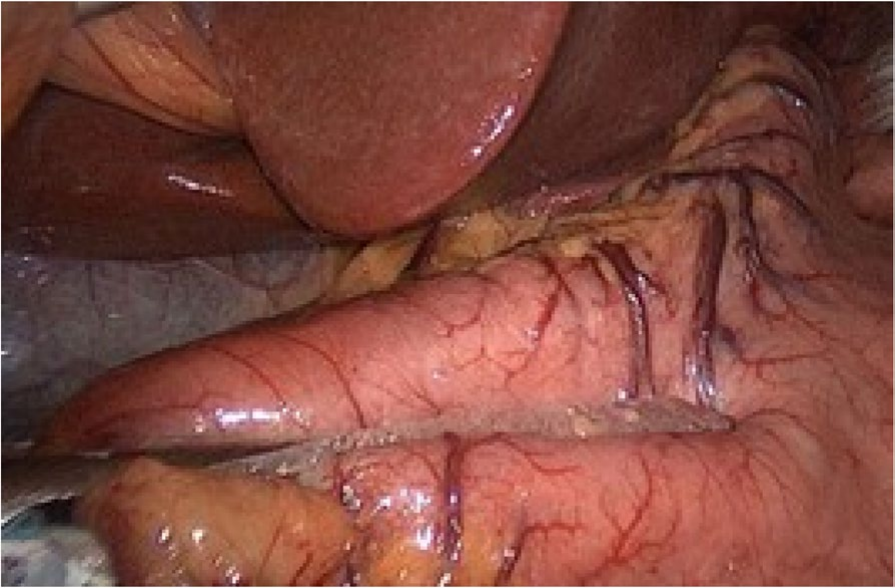
First staple fire in a sleeve gastrectomy

**Fig. 6:**
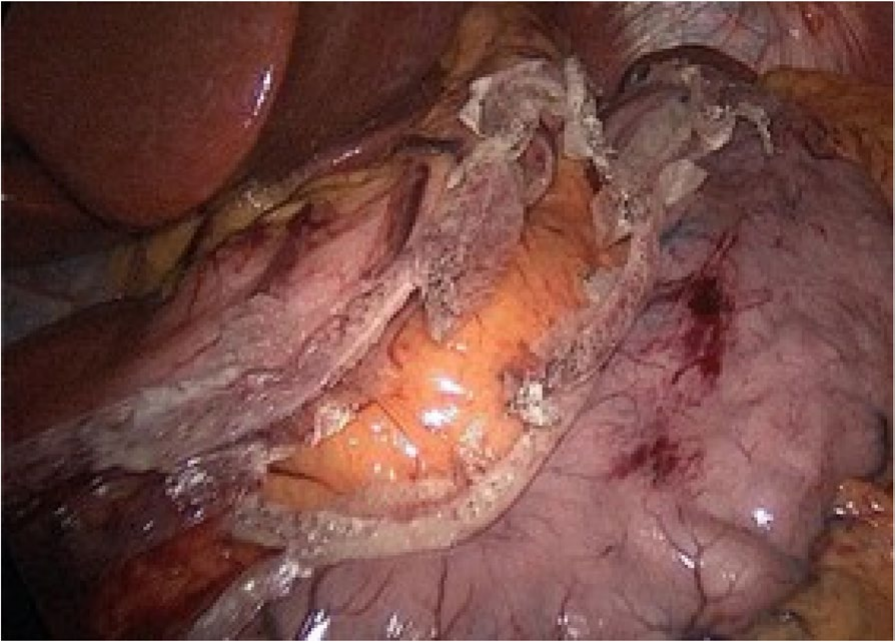
Completely divided sleeve after multiple fires

Dissection began approximately 6 cm from the pylorus, with complete mobilization of the greater curvature using an energy device (Fig. 4). Gastric transection was performed using a linear stapler along the bougie toward the angle of His, ensuring complete fundic resection (Figs. 5 and 6).

Staple line integrity was assessed intraoperatively using an air and methylene blue leak test. A drain was placed adjacent to the staple line, and all port sites ≥ 10 mm were closed to prevent herniation.

### Postoperative management

In the early postoperative period, all patients received third-generation cephalosporins as a local hospital policy, anticoagulants which were discontinued once the patient became ambulant, proton pump inhibitors 40 mg twice daily and continued over the period of 2 months, opioids, and antiemetics. On the second postoperative day, all patients underwent a water-soluble contrast study using Gastrografin to assess for potential leaks. By the third day, patients were started on oral fluids, provided they tolerated them well and the contrast study confirmed the absence of leakage. Patients were discharged within 48 to 72 h postoperatively, once they met the discharge criteria, which included no evidence of bleeding, leakage, or other complications.

### Follow-up

Patients underwent structured postoperative evaluations conducted by the study's bariatric surgeons at 2, 4, and 6 months following surgery. During each visit, weight loss progression was quantitatively assessed through body weight measurement and BMI calculation.

Active surveillance of new-onset GERD symptoms was performed using standardized clinical interviews. Patients were systematically questioned about characteristic reflux manifestations including heartburn, regurgitation, and vomiting. Those reporting symptoms suggestive of GERD were referred for prompt diagnostic upper gastrointestinal endoscopy.

As a protocol requirement, all participants underwent scheduled esophagogastroduodenoscopy at the 6-month interval. This endoscopic evaluation specifically assessed:

- Mucosal integrity of the distal esophagus
- Anatomical and inflammatory status of the sleeved stomach
- Presence of reflux-associated pathology including esophagitis, gastritis, ulceration, or stricture formation.

According to Los Angeles Classification, GERD is classified into four main grades based on the size and spread of mucosal breaks:

#### Grading criteria

- Grade A: One or more lesions less than or equal to 5 mm, not extending between top of mucosal folds.
- Grade B: One or more lesions more than 5 mm, not extending between top of mucosal folds.
- Grade C: Mucosal breaks extending between tops of two or more folds, involving < 75% of the circumference.
- Grade D: Mucosal breaks involving more than or equal 75% of the circumference [1].

**Table:** 

The Los Angeles (LA) classification system	Grade A	Grade B	Grade C	Grade D
Number of patients (of 16)	**9**	**5**	**2**	**0**
Percentage of patients				

**Table:** Relation of BMI and GERD improvement

16 patient	6 GERD improved	10 GERD persist
BMI	26 ‒ 37	

Symptom frequency and severity were objectively documented using a validated GerdQ- questionnaire. Supplementary Table 1. 

### Statistical analysis

Statistical analyses were conducted using IBM SPSS Statistics for Windows, Version 20.0 (IBM Corp., Armonk, NY, USA). All study data underwent systematic coding and tabulation prior to formal analysis. Continuous variables were expressed as mean ± standard deviation (SD) with corresponding ranges, while categorical variables were presented as frequency counts and percentages.

The normality distribution of quantitative data was formally evaluated using appropriate diagnostic measures (Kolmogorov–Smirnov/Shapiro–Wilk tests) to determine the suitability of parametric versus nonparametric statistical approaches. This assessment guided the selection of inferential methods to ensure rigorous interpretation of study outcomes.

## Results

### General characteristics

Table 1 shows that the total cohort (N = 60), 42 patients (70.0%) were male and 18 (30.0%) were female. Age distribution showed that 2 patients (3.3%) were younger than 30 years, 22 (36.7%) were 30–39 years, 28 (46.7%) were 40–49 years, and 8 (13.3%) were 50–59 years. Regarding preoperative BMI, 42 participants (70.0%) were categorized as class II obesity and 18 (30.0%) as class III obesity. Hypertension was present in 16 patients (26.7%), diabetes mellitus in 26 (43.3%), and hyperlipidemia in 38 (63.3%), whereas the onset of postoperative GERD was identified in 16 patients (26.7%) and absent in 44 (73.3%).

**Table 1:** General characteristics of the Participants, (N = 60)

Characteristics	Category	N (%)
Sex	Female	18 (30.0%)
	Male	42 (70.0%)
Age category	< 30	2 (3.3%)
	30–39	22 (36.7%)
	40–49	28 (46.7%)
	50–59	8 (13.3%)
BMI categories	Obesity II	42 (70.0%)
	Obesity III	18 (30.0%)
HTN at baseline	No	44 (73.3%)
	Yes	16 (26.7%)
DM at baseline	No	34 (56.7%)
	Yes	26 (43.3%)
Hyperlipidemia at baseline	No	22 (36.7%)
	Yes	38 (63.3%)

BMI categories: Obesity class I, 30.0–34.9 kg/m2; Obesity class II, 35.0–39.9 kg/m2

### GERD status postoperatively

When comparing characteristics between GERD-negative (n = 44) and GERD-positive (n = 16) patients in Table 2, sex distribution was similar: females 13 (29.5%) vs. 5 (31.3%) and males 31 (70.5%) vs. 11 (68.8%), respectively, with no significant association, χ2(1) = 0.33,*p* = 0.57*)*. Age categories also did not differ significantly between GERD groups (for < 30: 1/44 (2.3%) vs. 1/16 (6.3%); 30–39: 18/44 (40.9%) vs. 4/16 (25.0%); 40–49: 19/44 (43.2%) vs. 9/16 (56.3%); 50–59: 6/44 (13.6%) vs. 2/16 (12.5%); χ2(3) = 1.81,*p* = 0.61).

**Table 2:** Association between preoperative clinical characteristics and symptoms by GERD status

		GERD -ve N = 44 n (%)	GERD + ve N = 16 n (%)	*p* value
Preoperative characteristics				
Sex	Female	13 (29.5%)	5 (31.3%)	0.57
	Male	31 (70.5%)	11 (68.8%)	
Age category	< 30	1 (50.0%)	1 (50.0%)	0.61
	30–39	18 (81.8%)	4 (18.2%)	
	40–49	19 (67.9%)	9 (32.1%)	
	50–59	6 (75.0%)	2 (25.0%)	
BMI category	Obesity II	30 (71.4%)	12 (28.6%)	0.85
	Obesity III	14 (77.8%)	4 (22.2%)	
Postoperative characteristics				
Heartburn	No	44 (100.0%)	2 (12.5%)	< 0.001
	Yes	0 (0.0%)	14 (87.5%)	
Regurgitation	No	44 (100.0%)	6 (37.5%)	< 0.001
	Yes	0 (0.0%)	10 (62.5%)	
Epigastric or chest pain	No	44 (100.0%)	10 (62.5%)	< 0.001
	Yes	0 (0.0%)	6 (37.5%)	
Epigastric fullness	No	44 (100.0%)	10 (62.5%)	< 0.001
	Yes	0 (0.0%)	6 (37.5%)	
Dysphagia	No	44 (100.0%)	12 (75.0%)	0.001
	Yes	0 (0.0%)	4 (25.0%)	
Cough	No	44 (100.0%)	12 (75.0%)	0.001
	Yes	0 (0.0%)	4 (25.0%)	
Post-operative HTN	No	37 (84.1%)	13 (81.3%)	0.794
	Yes	7 (15.9%)	3 (18.8%)	
Post-operative DM	No	36 (81.8%)	12 (75.0%)	0.559
	Yes	8 (18.2%)	4 (25.0%)	
Post-operative hyperlipidemia	No	30 (68.2%)	14 (87.5%)	0.135
	Yes	14 (31.8%)	2 (12.5%)	

Likewise, BMI category (Obesity II vs. III) showed no significant association with GERD status (Obesity II: 30/44 (68.2%) vs. 12/16 (75.0%); Obesity III: 14/44 (31.8%) vs. 4/16 (25.0%); χ2(1) = 0.04,*p* = 0.85).​

In contrast, typical reflux symptoms were strongly associated with GERD. Heartburn was reported in 14 GERD-positive patients (87.5%) but in none of the GERD-negative patients (0.0%), χ2(1) = 50.22,*p* < 0.001. Regurgitation occurred in 10 GERD-positive patients (62.5%) vs. no GERD-negative (0.0%; χ2(1) = 33.00,*p* < 0.001). Epigastric or chest pain was present in 6 GERD-positive patients (37.5%) and absent in all GERD-negative patients (0.0%; χ2(1) = 18.33,*p* < 0.001). A very similar pattern was seen for epigastric fullness, with 6/16 (37.5%) vs. 0/44 (0.0%; χ2(1) = 18.33,*p* < 0.001). Dysphagia and cough were each reported in 4 GERD-positive patients (25.0%) and in no GERD-negative patients (0.0%), yielding significant associations (both χ2(1) = 11.79,*p* = 0.001*).* Post-operative hypertension (13/16 (81.3%) vs. 37/44 (84.1%); χ2(1) = 0.07,*p* = 0.79), diabetes (12/16 (75.0%) vs. 36/44 (81.8%); χ2(1) = 0.34,*p* = 0.56), and hyperlipidemia (2/16 (12.5%) vs. 14/44 (31.8%); χ2(1) = 2.24,*p* = 0.14) did not differ significantly between GERD groups.

### BMI change over 6 months

Mean BMI decreased progressively from 38.63 ± 1.53 kg/m^2^ pre-operatively to 36.13 ± 2.15 kg/m^2^ at 2 months, 34.37 ± 2.44 kg/m^2^ at 4 months, and 32.08 ± 3.21 kg/m^2^ at 6 months with a highly significant overall time effect (*p* < 0.001). Post-hoc pairwise comparisons showed significant decreases relative to what is present at 2 months (*p* < 0.001), 4 months (*p* < 0.001), and 6 months (*p* < 0.001), indicating a large and clinically meaningful weight loss that continued to accrue through 6 months.​ Table 3.

**Table 3:** BMI Reduction Over the Follow-up Period (N = 60)

BMI	Pre-operative	2 months	4 months	6 months	*P*-value^a^
	**N = 60**	**N = 60**	**N = 60**	**N = 60**	
Mean ± SD	38.63 ± 1.53	36.13 ± 2.15	34.37 ± 2.44	32.08 ± 3.21	< 0.001**
Range	36 ‒ 41	31 ‒ 40	29 ‒ 38	26 ‒ 37	
Pairwise*	**–**	**−4.2**^**b**^	**−8.3**^**c**^	**−12.6**^**d**^	
*P*-value	**–**	< 0.001 (HS)**	< 0.001 (HS)**	< 0.001 (HS)**	

^*^Pairwise Post-Hoc comparison

^**^P-value < 0.01: Highly significant

^a^Freidman test for related Groups

^b^2M vs Preoperative

^c^4M vs preoperative

^d^6M vs Preoperative

### Endoscopic findings among GERD patients

Among patients with GERD (N = 16), the prevalence of dilated cardia without esophagitis increased over time from 2 cases (12.5%) at 2 months to 4 (25.0%) at 4 months and 8 (50.0%) at 6 months, with a borderline-significant change (*p* = 0.05). In contrast, dilated cardia with esophagitis decreased from 12/16 (75.0%) at 2 months to 10/16 (62.5%) at 4 months and 6/16 (37.5%) at 6 months (*p* = 0.061), suggesting a clinically relevant but statistically non-significant improvement in esophagitis over time. Hiatal hernia remained stable at 2/16 (12.5%) at each assessment, with no significant temporal change (*p* = 1.00). Table 4.

**Table 4:** Endoscopic Findings for GERD patients at 2, 4, and 6 Months Following Sleeve Gastrectomy, (N = 16)

Endoscopy	2 months	4 months	6 months	*p* value*
Dilated cardia without esophagitis	2 (12.5%)	4 (25%)	8 (50%)	0.05
Dilated cardia with esophagitis	12 (75%)	10 (62.5%)	6 (37.5%)	0.061
Hiatal hernia	2 (12.5%)	2 (12.5%)	2 (12.5%)	1.0

^*^Q Cochrane test

## Discussion

Obesity represents a rapidly intensifying global health crisis, increasingly acknowledged as a paramount public health burden across developed and developing nations alike [8]. Its escalating prevalence is strongly associated with a diverse array of comorbidities, encompassing type 2 diabetes mellitus, hypertension, osteoarthritis, obstructive sleep apnea, non-alcoholic fatty liver disease, gastroesophageal reflux disease (GERD), and several malignancies.

Laparoscopic sleeve gastrectomy (LSG) has solidified its position as a highly effective bariatric intervention, consistently demonstrating significant and durable weight loss coupled with amelioration or resolution of numerous obesity-associated conditions [9]. However, its relationship with GERD continues to generate significant clinical controversy. While some investigations document notable improvement or resolution of pre-existing GERD post-LSG, others report the emergence of de novo GERD in a substantial minority of patients, with incidence rates varying considerably from 5 to 69% [10]. This heterogeneity underscores the physiological complexity of post-LSG reflux and necessitates further exploration of patient selection, technical factors, and long-term sequelae.

Our results corroborate previous research affirming LSG's efficacy in achieving marked short-term weight reduction. The mean BMI declined from 38.63 kg/m^2^ preoperatively to 32.08 kg/m^2^ at six months postoperatively, aligning with established data on early postoperative weight loss efficacy following LSG [11, 12].

Beyond weight reduction, significant resolution of obesity-related comorbidities, including hypertension, type 2 diabetes mellitus, and hyperlipidemia, were observed. Nevertheless, new-onset GERD symptoms developed in 26.67% (16/60) of patients, while 73.33% (44 patients) remained symptom-free. This incidence falls within the spectrum reported in prior studies, where postoperative GERD rates range from 0% to 34.9% [13]. Illustrating this variability, Arias et al. [14] documented a GERD rate of 2.1%, and Kehagias et al. [15] reported 7.4%. Conversely, Braghetto et al. [16] observed a higher incidence of 27.5%, and Tai et al. [17] reported 37.4%. Del Genio et al. [18], conversely, found no significant LSG impact on GERD at 13-month follow-up. However, it is important to acknowledge that postoperative proton pump inhibitor use may represent a confounding factor affecting symptom reporting and clinical outcomes.

The pathophysiology of post-LSG GERD is frequently linked to anatomical alterations inherent to the procedure, such as resection of the angle of His and modifications in lower esophageal sphincter (LES) function. Comprehensive postoperative endoscopic evaluation remains relatively sparse in the literature, though Braghetto et al. [16] specifically reported instances of erosive esophagitis following the procedure.

The First International Consensus Summit for Sleeve Gastrectomy documented postoperative GERD in 4.7% ± 8.9% of patients (range: 0–36%) [12]. Reported rates of reflux symptoms post-LSG specifically vary between 2.8% and 13% [19–22]. Almogy et al. [23] similarly posited that LSG may induce or aggravate GERD, noting a 13% incidence necessitating proton pump inhibitor (PPI) therapy. Nocca et al. [20] identified GERD in 11.8% of their LSG cohort. The Second International Consensus Summit reported a mean GERD incidence after LSG of 6.5% ± 14.3%, with an exceptionally wide reported range (0% to 83%).

Multiple physiological and anatomical mechanisms potentially explain post-LSG GERD development. Proposed factors include diminished LES pressure secondary to periesophageal dissection, obliteration of the angle of His, reduced gastric compliance, elevated intragastric pressure, and perturbed gastric emptying. Counteracting these effects, improvements in gastric motility and sustained weight loss may confer benefits over time. Some evidence suggests potential long-term restoration of gastric compliance and the angle of His, possibly facilitating GERD symptom resolution approximately three years postoperatively.

Surgical modifications have been proposed to mitigate post-LSG GERD. Fedenko and Evdoshenko [24] described an antireflux sleeve gastroplasty integrating vertical gastroplasty with Nissen fundoplication. Alexander et al. [25] introduced a banded SG utilizing a dermal graft to forestall sleeve dilation and alleviate GERD. Korwar et al. [26] combined LSG with concurrent laparoscopic hiatal hernia repair, demonstrating efficacy in reflux control and weight loss maintenance.

Santoro [11] hypothesized that SG might be inherently refluxogenic due to factors like undiagnosed hiatal hernia, division of the phrenoesophageal ligament, a preserved pylorus coupled with a narrow gastric tube, sleeve migration, incisural narrowing, or fundic regrowth forming a neostomach.

Our investigation specifically focused on de novo GERD development in patients devoid of preoperative reflux symptoms. Prior studies yield conflicting findings on this issue. Authors such as Arias et al. [14], Liu et al. [19], and Nocca et al. [20] documented increased GERD prevalence post-SG. In contrast, Cottam et al. [27] reported a reduction in GERD incidence following surgery.

Among studies reporting overall GERD reduction, authors observed that while pre-existing symptoms often improved, new postoperative GERD cases still arose. Himpens et al. [28], for instance, noted resolution in 75% of patients with preoperative GERD but also identified a 21.8% incidence of new GERD at one year. Melissas et al. [29] similarly found reduced preoperative GERD burden but documented two new postoperative cases, though statistical significance was not consistently reported. Notably, Himpens et al. [28] observed that while GERD symptoms frequently worsened initially, improvement typically occurred by two to three years postoperatively.

Several studies specifically examined postoperative GERD prevalence [30, 31]. In our cohort, all 16 symptomatic patients received PPI therapy. By the six-month follow-up, symptomatic improvement was evident in 6 patients (37.5%), while 10 patients (62.5%) reported persistent symptoms.

While some surgeons regard hiatal hernia or pre-existing GERD as contraindications to SG, our perspective differs. The anatomical and physiological consequences of SG on GERD remain actively debated. Hamoui et al. [31] cautioned that alterations to the angle of His and disruption of anatomical barriers during SG might exacerbate GERD, advising prudence in selecting patients with known GERD. Conversely, Melissas et al. [29] reported accelerated gastric emptying after SG in both short and long terms, attributing worsened GERD primarily to surgical dissection near the cardia.

In selected patients, particularly those with risk factors for reflux or anatomical predisposition, the addition of anti-reflux procedures or concomitant hiatal hernia repair during sleeve gastrectomy has been proposed as a strategy to mitigate postoperative GERD. The rationale for this approach is based on the potential restoration of the anti-reflux barrier, including reinforcement of the gastroesophageal junction and reduction of intragastric pressure-related reflux. Several studies have suggested that addressing hiatal defects or combining sleeve gastrectomy with anti-reflux techniques may improve reflux outcomes, although the evidence remains heterogeneous and further studies are required to establish standardized indications [19].

Recent physiological evidence has provided further insight into strategies aimed at reducing postoperative reflux following sleeve gastrectomy. High-resolution manometry studies have demonstrated that combining sleeve gastrectomy with anti-reflux procedures, such as Rossetti fundoplication, may lead to improved lower esophageal sphincter (LES) pressure and enhanced esophagogastric junction competence. These findings suggest a potential protective mechanism against postoperative reflux and may offer a valuable surgical modification in selected patients at higher risk of GERD. However, further large-scale and long-term studies are required to confirm the clinical efficacy and safety of these combined approaches [32, 33].

## Conclusion

This study demonstrates that de novo GERD may develop in a subset of patients following laparoscopic sleeve gastrectomy. While LSG remains an effective bariatric procedure for weight loss and metabolic improvement, it may be associated with an increased risk of postoperative reflux in certain patients. Therefore, careful preoperative evaluation and individualized patient selection are essential, particularly in those with potential risk factors for reflux. Further large-scale studies with longer follow-up and objective diagnostic tools are required to better clarify the relationship between LSG and GERD.

### Limitations

The study is limited by its small sample size, short six-month follow-up, and reliance on clinical symptoms without routine endoscopic or pH testing, potentially underestimating GERD. Uncontrolled variables like diet, adherence, and surgical variation, along with the absence of a control group, also restrict broader applicability.

### Recommendations

Routine preoperative endoscopy is essential to identify conditions like hiatal hernia or Barrett’s esophagus that may contraindicate sleeve gastrectomy, favoring Roux-en-Y gastric bypass instead. GERD patients require individualized assessment. Longer follow-up and future studies using objective diagnostics and evaluating surgical technique variations are needed to clarify reflux outcomes.

## Supplementary Information

Supplementary Material 1.

## Data Availability

The datasets generated and analyzed during the current study are not publicly available due to ethical and confidentiality considerations involving patient data but are available from the corresponding author upon reasonable request.
