# Supplementary figures and images for: Short term detection of de novo gastroesophageal reflux disease after laparoscopic sleeve gastrectomy
Source: BMC Gastroenterol. 2026 Jul 14;26:446. doi: 10.1186/s12876-026-05018-7 (PMC13371026; doi:10.1186/s12876-026-05018-7)

Supplementary Table.1


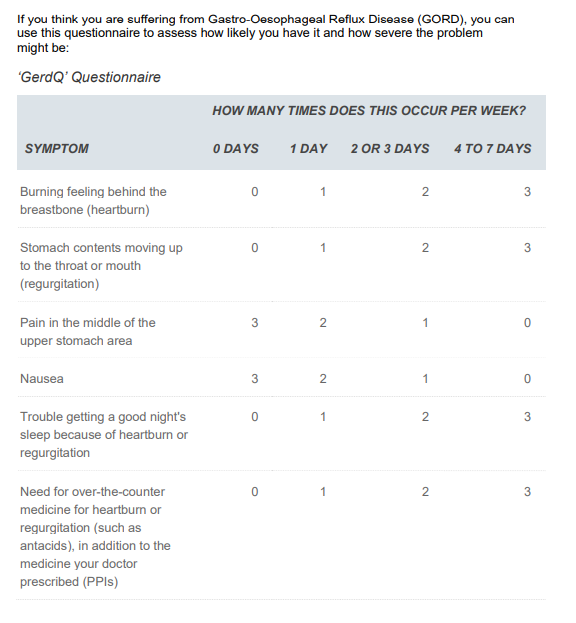


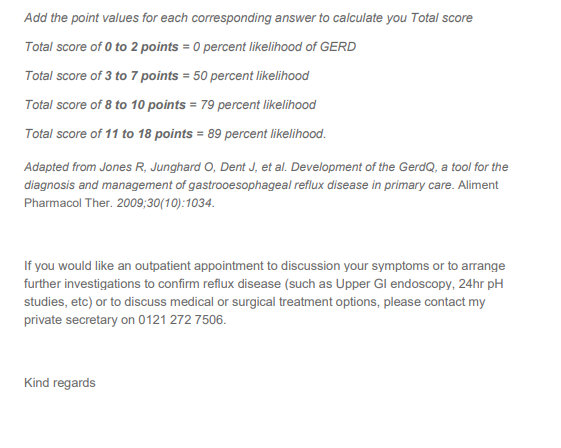

Supplement: Supplementary file 1 — Supplementary Material 1. [file 12876_2026_5018_MOESM1_ESM.docx]
